# HbMADS4, a MADS-box Transcription Factor from *Hevea brasiliensis*, Negatively Regulates *HbSRPP*

**DOI:** 10.3389/fpls.2016.01709

**Published:** 2016-11-15

**Authors:** Hui-Liang Li, Li-Ran Wei, Dong Guo, Ying Wang, Jia-Hong Zhu, Xiong-Ting Chen, Shi-Qing Peng

**Affiliations:** Key Laboratory of Biology and Genetic Resources of Tropical Crops, Ministry of Agriculture, Institute of Tropical Bioscience and Biotechnology, Chinese Academy of Tropical Agricultural SciencesHaikou, China

**Keywords:** *Hevea brasiliensis*, small rubber particle protein, promoter, MADS-box transcription factor, negative regulator, natural rubber

## Abstract

In plants MADS-box transcription factors (TFs) play important roles in growth and development. However, no plant MADS-box gene has been identified to have a function related to secondary metabolites regulation. Here, a MADS-box TF gene, designated as *HbMADS4*, was isolated from *Hevea brasiliensis* by the yeast one-hybrid experiment to screen the latex cDNA library using the promoter of the gene encoding *H. brasiliensis* small rubber particle protein (HbSRPP) as bait. *HbMADS4* was 984-bp containing 633-bp open reading frame encoding a deduced protein of 230 amino acid residues with a typical conserved MADS-box motif at the N terminus. *HbMADS4* was preferentially expressed in the latex, but little expression was detected in the leaves, flowers, and roots. Its expression was inducible by methyl jasmonate and ethylene. Furthermore, transient over-expression and over-expression of HbMADS4 in transgenic tobacco plants significantly suppressed the activity of the *HbSRP* promoter. Altogether, it is proposed that HbMADS4 is a negative regulator of *HbSRPP* which participates in the biosynthesis of natural rubber.

## Introduction

The MADS-box transcription factors (TFs) family is found throughout eukaryotes (Theissen et al., 2000). The MADS-box TF proteins contain a typical conserved MADS-box motif and dimerization domain at the N-terminal. The MADS-box TFs family contains much more number of members in plants than in other eukaryotes (Grimplet et al., 2016). In plants, MADS-box TFs are best known as regulators of reproductive development, such as flowering induction, flower development, or fruit development (Theissen et al., 2000; Parenicová et al., 2003; Adamczyk et al., 2007; Arora et al., 2007; Itkin et al., 2009; Smaczniak et al., 2012a,b; Puig et al., 2013). MADS-box TFs have also been found extensively in vegetative tissues, embryo, root, trichome, etc, suggesting their diverse functions in plant growth and development processes (Alvarez-Buylla et al., 2000; Ku et al., 2008). MADS-box TFs are also involved in biotic or abiotic stress response regulation (Arora et al., 2007; Khong et al., 2015). However, no plant MADS-box gene has been identified to have a function related to secondary metabolites regulation.

*Hevea brasiliensis* Müll. Arg (rubber tree) is the only plant widely cultivated to produce natural rubber (NR) (van Beilen and Poirier, 2007). NR (a *cis* 1, 4-polyisoprene) biosynthesis occurs on rubber particles suspended in rubber tree laticifers (d’Auzac et al., 1989). Latex (cytoplasmic content of laticifers) contants 30–50% *cis*-polyisoprene which is synthetized through the mevalonate pathway (Chow et al., 2007, 2012; Sando et al., 2008). The NR biosynthesis is a typical isoprenoid secondary metabolism which is similar to the isoprenoid biosynthesis of other plants (Chow et al., 2007). NR is generated by three main steps including initiation, elongation and termination (Puskas et al., 2006). The prenyltransferase, rubber elongation factor and small rubber particle protein play important roles during elongation, giving rise to the long chains of *cis*-polyisoprene (Dennis and Light, 1989; Oh et al., 1999; Asawatreratanakul et al., 2003; Berthelot et al., 2012). *H. brasiliensis* small rubber particle protein (HbSRPP) is a major latex protein and obviously participates in NR biosynthesis (Oh et al., 1999; Berthelot et al., 2012). In *H. brasiliensis*, the general metabolic pathway leading to rubber biosynthesis is now clear (Chow et al., 2007; Sando et al., 2008), but the molecular regulation of NR rubber in *H. brasiliensis* is limited (Wang et al., 2013). Therefore, the identification and functional study of regulation of NR biosynthesis-related gene may elucidate the molecular mechanisms of NR biosynthesis in rubber tree. Here, we report identification and functional analysis of the rubber tree *HbMADS4* that encodes a MADS-box TF. HbMADS4 activated the *HbSRPP* promoter in yeast. Gel shift assay and co-transfection results revealed HbMADS4 and *HbSRPP* promoter interaction and supression of *HbSRPP* expression, indicating that HbMADS4 maybe a negative transcription regulator of *HbSRPP* involved in NR biosynthesis.

## Materials and Methods

### Plant Material

*Hevea brasiliensis* cultivar CATAS7-33-97 was planted in the experimental plantation of the Chinese Academy of Tropical Agriculture Sciences (Danzhou Hainan, PR, China). The epicormic shoots were treated by methyl jasmonate (MeJA) and Ethylene (ET) as described previously (Hao and Wu, 2000). Latex, roots, leaves, and flowers were collected and then immediately frozen into liquid nitrogen.

### Yeast One-Hybrid

Latex RNA was isolated as described previously (Tang et al., 2007). Latex cDNAs synthesis was performed according to the instructions of cDNA Synthesis Kit (Fermentas). The promoter of *HbSRPP* was amplified by PCR with the primers (Guo et al., 2014). The bait vector was constructed by inserting the promoter of *HbSRPP* into pAbAi vector (Clontech) at the site of *Hind* III/*Xho* I. Latex cDNAs, the *Sma* I-linearized pGADT7-Rec prey vector and the bait vector were introduced into the yeast strain Y1HGold (Clontech). The transformed yeast cells were grown on SD/-Leu selective medium containing 450 ng/ml Aureobasidin A (AbA) at 30 for 3 d. The positive colonies were further analyzed by colony PCR and sequence analysis.

To confirm the binding specificity of HbMADS4 with the *HbSRPP, HbMADS4* was amplified by PCR from latex cDNA. *HbMADS4* was ligated into the pGADT7-Rec vector, generating pGADT7-HbMADS4. pGADT7-HbMADS4 and p*HbSRPP*-AbAi were co-transformed into the Y1HGold yeast strain. pGADT7-Rec53+p53-AbAi, pAbAi-*HbSRPP*, pGADT7-HbMADS4 and pGADT7-HbMADS4 + pAbAi were used as control. Transformed yeast cells were cultured on SD/-Leu selective medium containing 450 ng/ml AbA for 3 d at 30°C.

### Phylogenetic Analysis

A total of 38 MADS-box IF sequences from other plants were downloaded from NCBI database. The phylogenetic tree was constructed by employing MEGA version 4.0 using the neighbor-joining method with 1000 bootstrap replicates.

### Quantitative PCR (qPCR)

Total RNA was isolated as described previously (Tang et al., 2007). First strand cDNA was synthesized using cDNA synthesis kit according to the manufacturer’s instruction (Fermentas). Quantitative PCR (qPCR) was performed according to Wang’s method (Wang et al., 2013). The primer pairs used for the *HbMADS4* were 5′-CACAGCTGTATGTACTTACCTATC-3′) and MR (5′-CACAGCTGT ATGTACTTACCTATC-3′). *HbACT7* was used as internal control as recommended previously (Li et al., 2014). The details of experimental manipulations and data analysis were as described previously (Wang et al., 2013).

### Subcellular Localization

The *HbMADS4* ORF was amplified by PCR using the following primer pairs F1 (ACGCC ATGGTATGACAAGGCAGAAAATCCAGA) and R1 (ATTGAGATCTATCTGGGAAGGGTAA CCCCAAT), which contained *Nco* I/*Bgl* II site (underlined), respectively. The PCR products were ligated into the pCAMBIA1302 vector at the site of *Nco* I /*Bgl* II, generating *CaMV*35S::HbMADS4-GFP. *CaMV*35S::HbMADS4-GFP and pCAMBIA1302 were transformed into onion epidermal cells vis *Agrobacterium*-mediated transformation. The introduced cells were cultured on MS medium in darkness at 25°C for 24 h and then visualized by confocal microscopy.

### Recombinant HbMADS4 Protein and Purification of HbMADS4

The coding regions of *HbMADS4* was amplified with the following primer pairs: 5′- ACGTG GATCCATGACAAGGCAGAAAATCCAGA 3′ (with the *Bam*HI site underlined) and 5′-AT TGCTCGAGTCAATCTGGGAAGGGTAACCCC-3′ (with the *Xho*I site underlined). PCR products were ligateded into pET28a (+) vector (Novagen, Madison, WI, USA) at the site of *Bam*HI/*Xho*I sites. The constructed plasmid was introduced into *Escherichia coli* strain *Rosetta* (DE3). Protein biosynthesis was induced with 1.0 mM isopropyl-β-D-thiogalactopyranoside (IPTG) at 37°C. After inducting for 4 h, bacteria were harvested and resuspended in the extraction buffer containing 200 mM Tris/HCl pH 8.0, 5 mM 2-mercaptoethanol, 0.1% Triton X-100 and lysozyme (100 μg ml^-1^), and incubated at 30°C for 30 min. The extract was centrifuged at 12000 g for 30 min at 4°C. The supernatant was precipitated and purified on a HiTrap affinity column (GE Healthcare).

### Electrophoretic Mobility Shift Assay (EMSA)

Electrophoretic Mobility Shift Assay was performed using the Electrophoretic Mobility Shift Assay kit (Invitrogen, Carlsbad, CA, USA). The DNA-protein binding reaction was performed by incubating purified HbMADS4 with double-stranded *HbSRPP* promoter nucleotides at room temperature for 30 min, and then analyzed by polyacrylamide gel electrophoresis. The gel was stained with SYBR Green EMSA stain for visualizing DNA, the same gel was stained with SYPRO Ruby EMSA stain for monitoring protein.

### Dual-Luciferase (Dual-LUC) Assay

The assay was performed according to Hellens’s method (Hellens et al., 2005). In brief, the *HbSRPP* promoter was cloned into pGreen-II vector, generating pGreen-HbSRPP. *HbMADS4* was inserted pGreen-II62SK, generating pGreenII62SK-HbMADS4. Two constructs were introduced into *Agrobacterium tumefaciens* (strain GV3101). The introduced *Agrobacterium* cells harboring pGreenII-HbSRPP were mixed with the *Agrobacterium* strains harboring pGreenII62SK-HbMADS4, in a volume ratio of 1:2. The *Agrobacterium* mixtures were infiltrated into the abaxial side of tobacco leaves. After culturing for 3 days, total protein was extracted from the infected area. The fluorescent values of LUC and REN were detected using the Dual-Luciferase Reporter Assay System (Promega) following the manufacture’s manual. The value of LUC was normalized to that of REN. Three biological repeats were measured.

### Plant Transformation and GUS Assay

The promoter of *HbSRPP* was inserted into the pCAMBIA1381 vector at the site of *Bam*H I /*Hin*d III, generating *pHbSRPP*::*GUS* (PH). The construct was introduced into the *A. tumefaciens* strain GV3103. Tobacco transformation was performed using a leaf disc vis *Agrobacterium*-mediated method (Horsch et al., 1985). The T_0_ transgenic plants were selected hygromycin resistance and GUS histochemical staining. The T_1_ seedings (HP) were grown on MS medium containing hygromycin. For the effector construct, the *HbMADS4* cDNA was inserted into the pBI121 vector at a site of *Xba* I/*Sac* I, under the control of the CaMV *35S* promoter. The effector was transformed into HP by *Agrobacterium* -mediated method. Co-transformed plants (CTPs) were selected by hygromycin and kanamycin resistance, further tested by RT-PCR. *HbMADS4* was amplified with a specific primer pairs P1 (5′-CTGCAACTGGGAAGCTCTTTGAGT-3′) and P2 (5′-GCAGCTCAAGAGAAG GTTGATCT-3′). The *NtACT* was used as an internal control parallel in the reactions. *NtACT* was amplified with *a* specific primers AF (5′-TGTCAGCAACTGGGACGATATGG-3′) and AR (5′- GA GTCATCTTCTCTCTGTTGGC-3′). CTPs were separately assayed for GUS activity by fluorometry as described previously (Jefferson, 1987). Three biological repeats were measured. Protein content was determined using the Bradford protein assay (Bradford, 1976). Data were subjected to ANOVA.

## Results

### Cloning of *HbMADS4*

**FIGURE 1:**
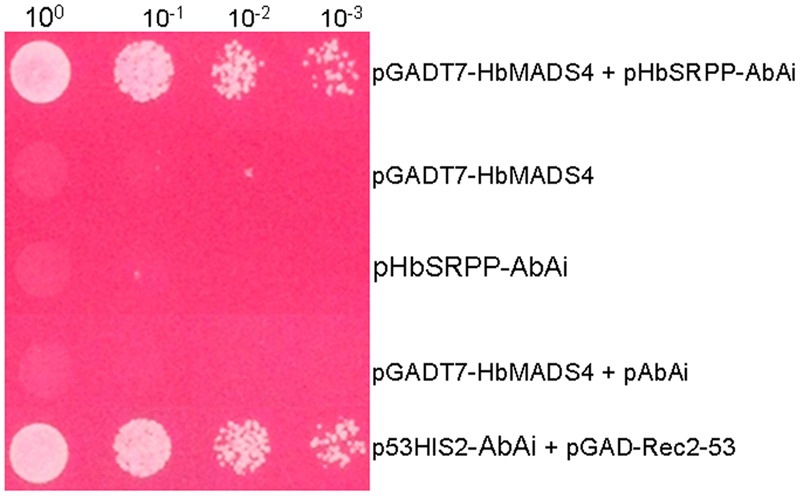
**Activation of *HbSRPP* promoter in yeast by HbMADS4.** Yeast cells carrying pGADT7- HbMADS4+p*HbSRPP*-AbAi, pGADT7-Rec53+p53-AbAi, p*HbSRPP*-AbAi, pGADT7-HbMADS4, and pGADT7-HbMADS4+pAbAi were grown in SD/-Leu selective medium containing 450 ng/ml AbA for 3 days at 30°C.

In order to screen novel TFs which regulate *HbSRPP*, we performed yeast one-hybrid assay with the rubber tree latex cDNA library using the *HbSRPP* promoter as bait. 48 positive colonies were obtained and sequenced. BLAST analysis showed one cDNA (designated as *HbMADS4*, GenBank accession No. KX100586) encoding a MADS-box protein. The binding specificity of the MADS-box protein with the *HbSRPP* promoter was determined by the one-to-one interaction analysis. As shown in **Figure 1**, only the yeast clones harboring *HbMADS4* and pAbAi-*HbSRPP* or positive control could grow on the SD/-Leu selective medium containing 450 ng/ml AbA, indicating that HbMADS4 bound to the *HbSRPP* promoter and activated transcription in yeast.

### Molecular Characterization of *HbMADS4*

**FIGURE 2:**
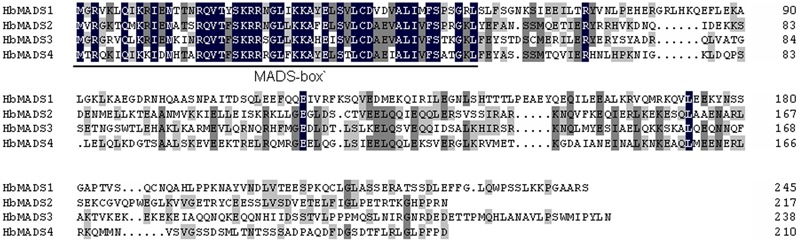
**Alignment of HbMADS4 and other rubber tree MADS-box transcription factors (TFs).** The amino acid sequence of HbMADS4 was aligned with HbMADS1 (GenBank accession no. GU142913), HbMADS2 (GU142914), and HbMADS3 (GU142915). Black and gray shadings indicate conserved amino acid residues.

**FIGURE 3:**
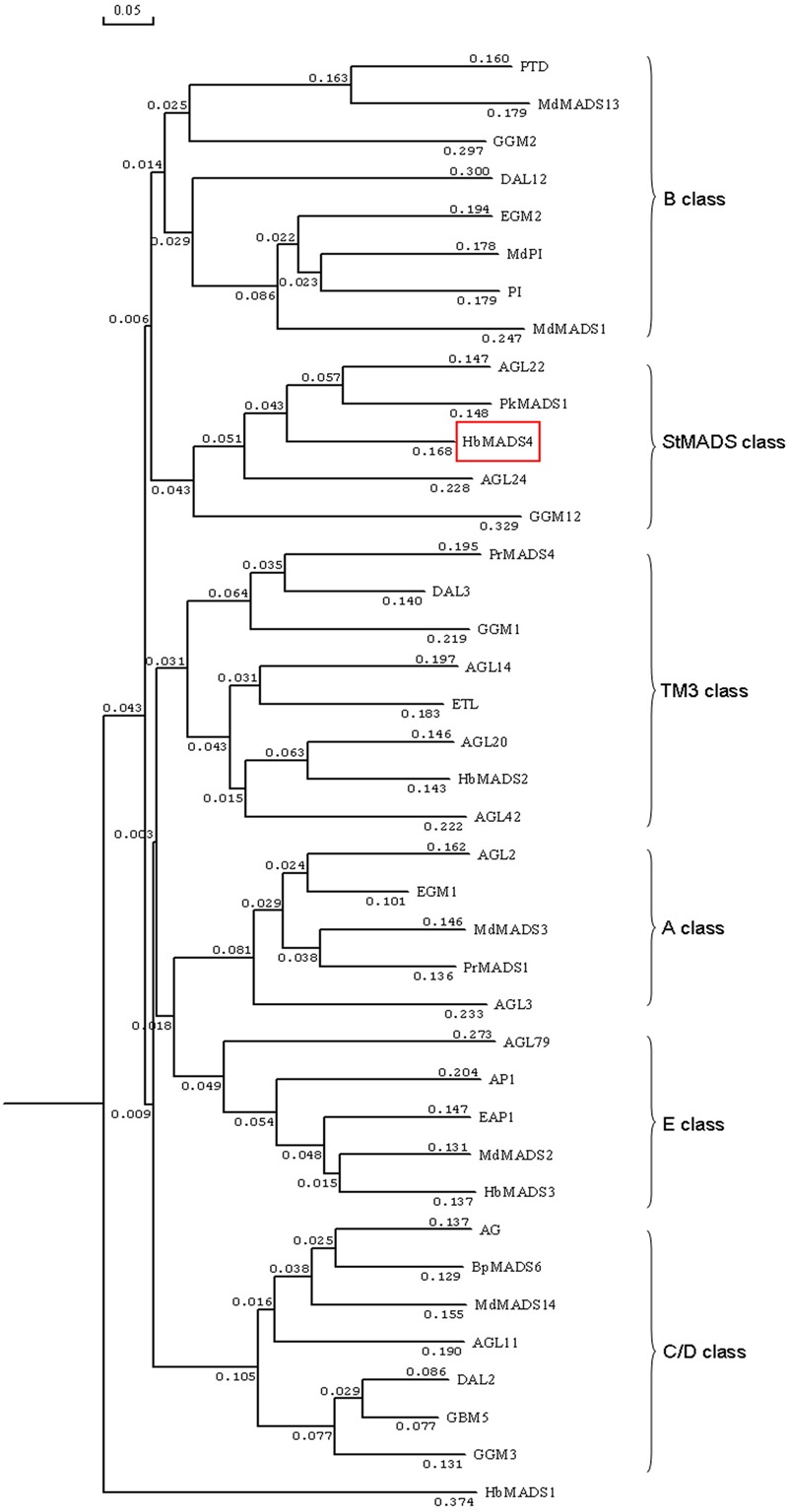
**Phylogenetic analysis of HbMADS4 with other MADS-box TFs by MEGA version 2.1 from CLUSTAL W alignments.** The neighbor-joining method was used to construct the tree. The MADS-box TFs used in the evolutionary analysis are retrieved from Genbank including AG (*Arabidopsis thaliana*, P17839), AGL2 (*A. thaliana*, P29382), AGL3 (*A. thaliana*, 30678072), AGL11 (*A. thaliana*, GI12229648), AGL14 (*A. thaliana*, Q38838), AGL20 (*A. thaliana*, O64645), AGL22 (*A. thaliana*, Q9FVC1), AGL24 (*A. thaliana*,CAB79364), AGL42(*A. thaliana* AAN52777), AGL79 (*A. thaliana*, AAN52802), AP1 (*A. thaliana*, S27109), BpMADS6 (*Betula pendula*, CAA67968), DAL2 (*Picea abies*, S51934), DAL3 (*P. abies*, S51936), DAL12 (*P. abies*, AAF18375), EAP1 (*Eucalyptus globules*, AAG24909), EGM1(*E. grandis*, AAC78282), EGM2(*E. grandis*, AF029976), ETL(*E. globulus*, AAD16052), GBM5 (*Ginkgo biloba*, AAM76208), GGM1 (*Gnetum gnemon*, CAB44447), GGM2 (*G. gnemon*, CAB44448), GGM3 (*G. gnemon*, CAB44449), GGM12 (*G. gnemon*, CAB44458), HbMADS1 (*Hevea brasiliensis*,GU142913), HbMADS2 (*H. brasiliensis*, GU142914), HbMADS3 (*H. brasiliensis*,GU142915), HbMADS4 (*H. brasiliensis*, KX100586), MdMADS1 (*Malus domestica*, AAC25922), MdMADS2 (*M. domestica*, AAC83170), MdMADS3 (*M. domestica*, AAD51422), MdMADS13 (*M. domestica*, CAC80856), MdMADS14 (*M. domestica*, CAC80857), MdPI (*M. domestica* CAC28021), PkMADS1 (*Paulownia kawakamii*, AAF22455), PI (*A. thaliana*, NP_197524), PTD (*Populus trichocarpa*, AAC13695), PrMADS1 (*Pinus radiata*, T09569), and PrMADS4 (*P. radiate*, AAB80807).

*HbMADS4* was 984-bp containing 633-bp open reading frame encoding a deduced protein of 230 amino acid residues protein with a predicted molecular mass of 23.71 kD. The deduced HbMADS4 protein contained a MADS-box domain which is typical for the MADS-box TFs (**Figure 2**). However, the entire HbMADS4 protein sequence has limited identity with other MADS-box protein reported in rubber tree (**Figure 2**). HbMADS4 should be a new member of the rubber tree MADS-box family. HbMADS4 amino acid sequences and MADS-box from other different species were compared and a phylogenetic tree showed that HbMADS4 is members of the StMADS class subfamily (**Figure 3**).

### Analysis of *HbMADS4* Expressions

**FIGURE 4:**
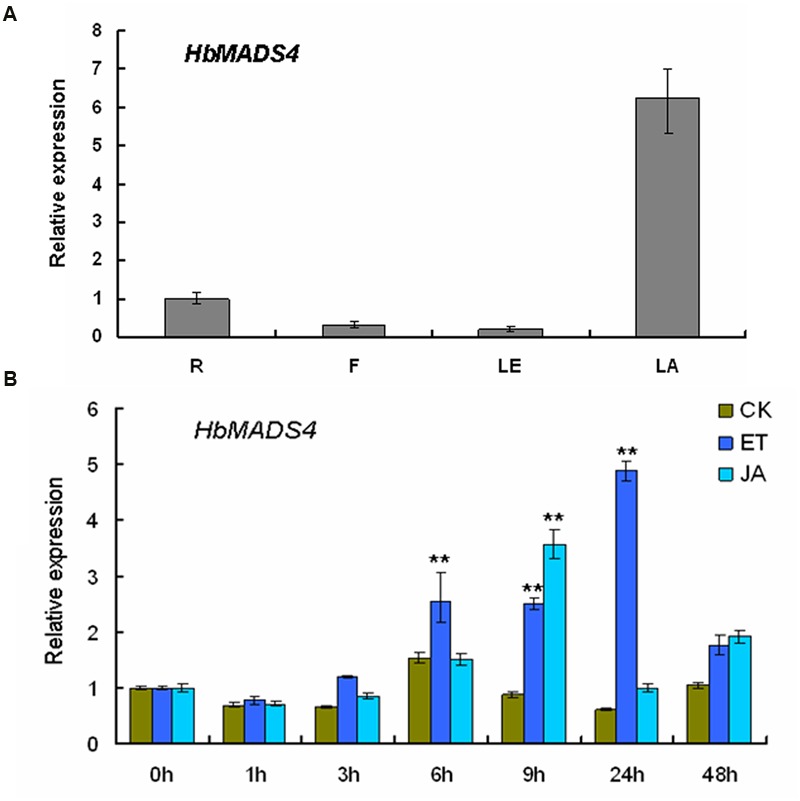
**Transcription patterns of *HbMADS4*. (A)** Differential expression of *HbMADS4* in different rubber tree. R, roots; B, bark; LE, leaves; LA, latex. **(B)** Expression patterns of *HbMADS4* respond to JA and ET treatment. The y axis is the scale of the relative transcript abundance level. The x axis is the time course of JA and ET treatment. An average of three independent biological replicates of each time was performed. Data are presented as mean ± SE (*n* = 3). The significant difference was assessed by ANOVA (two asterisks corresponding to *P* < 0.01).

The *HbMADS4* transcript levels were detected in different rubber tree tissues by qPCR. qPCR detected a signification level of *HbMADS4* in the latex, and the level was low in the leaves, flowers, and roots (**Figure 4A**). Signaling pathways, especially those of MeJA and ET are actively implicated in the regulation of latex regeneration (Zhu and Zhang, 2009; Duan et al., 2010; Tang et al., 2013). To examine the expression patterns of the *HbMADS4* in response to MeJA and ET, the rubber tree shoots were treated by MeJA or ET, respectively. As shown in **Figure 4B**, JA markedly up-regulated *HbMADS4* expression. The expression of *HbMADS4* increased and reached the highest level at 9 h, and then decreased at 24 h. The expression of *HbMADS4* was also induced by ET. The expression of *HbMADS4* began to increase at 6 h and reached the highest level at 24 h, and then remarkably decreased at 48 h. (**Figure 4B**).

### Subcellular Localization of HbMADS4

**FIGURE 5:**
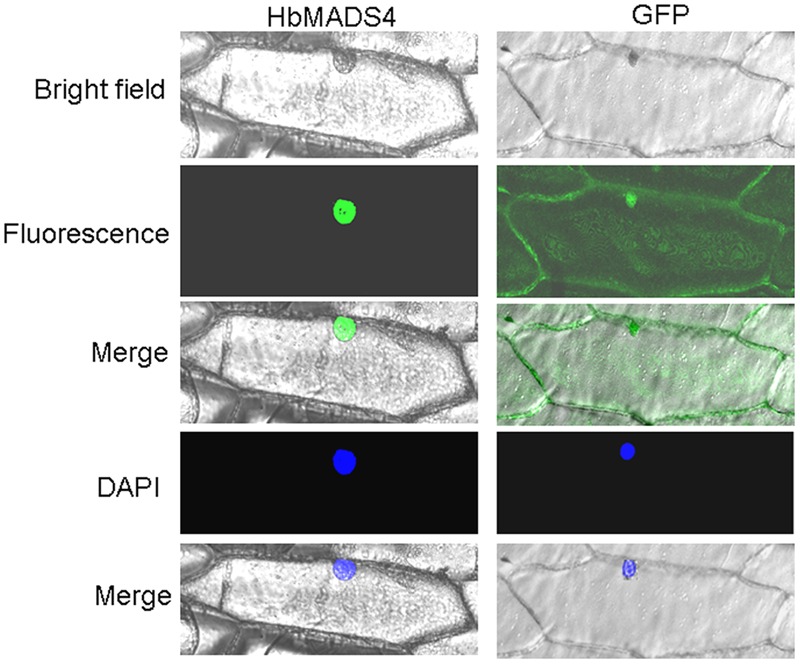
**Nuclear localization of HbMADS4.** The right panel, the corresponding fluorescence, bright field, merged fluorescence image, and DAPI image of GFP control; the left panel, the corresponding fluorescence, bright field, merged fluorescence image, and DAPI image of HbMADS4-GFP.

To study the subcelluar location of the HbMADS4 protein, the green fluorescent protein (GFP) was used as a reporter. *GFP* was fuse in frame to 5′ end of the ORF of *HbMADS4* in the pCAMBIA1302, resulting construct GFP-HbMADS4. GFP-HbMADS4 or pCAMBIA1302 was introduced into onion epidermal cells. As shown in **Figure 5**, GFP-HbMADS4 fusion protein was located in the nuclei of onion epidermal cell. In contrast, in the control GFP fluorescence was observed throughout the onion epidermal cell. These results indicate that HbMADS4 is a nuclear-localized protein.

### HbMADS4 Can Interact with the *HbSRPP* Promoter *In Vitro*

**FIGURE 6:**
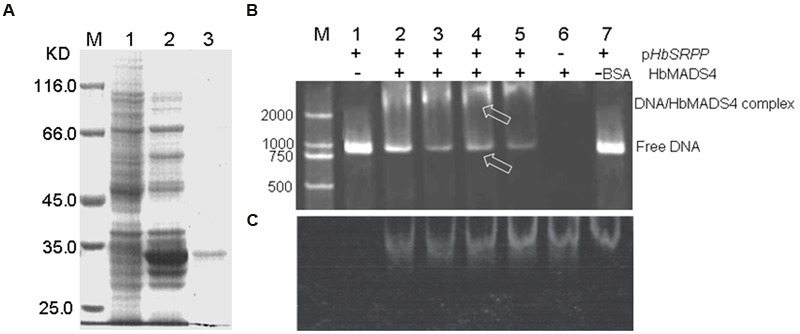
**HbMADS4 binding to the promoter of *HbSRPP* as analyzed by EMSA. (A)** Over-expression of *HbMADS4* in *Escherichia. coli*. Expression vector (pET- HbMADS4) was constructed in which the fusion protein was driven by the T7 promoter, made IPTG-inducible, and transformed into *E. coli* BL21 (DE3). Expression was induced by the addition of 0.2 mM IPTG, and total cell proteins were analyzed after 5 h by SDSPAGE. M molecular markers; 1. *E. coli* cells harboring pET-HbMADS4 not induced; 2, *E. coli* cells harboring pET-HbMADS4 after 5 h of induction; 3. the purified HbMADS4 fusion protein by *E. coli* cells harboring pET-HbMADS4 after 5 h of induction. **(B)** The *HbSRPP* promoter with HbMADS4 protein stained with SYBR green EMA for visualizing DNA. **(C)** The same gel as in **(B)** stained with SYPRO Ruby EMSA for visualizing protein. M DNA maker (DL2000). Lane 1. the promoter of *HbSRPP* DNA only; Lanes. 2–5 the promoter of *HbSRPP* DNA with increasing amounts of HbMADS4 protein (50, 100, 150, and 200 ng); Lane 6. 200 ng HbMADS4 protein only; Lane 7. 200 ng of bovine serum albumin (BSA) and the promoter of *HbSRPP* DNA, respectively. The arrows indicated the HbMADS4-DNA complex or free DNA.

To investigate whether HbMADS4 binds to the *HbSRPP* promoter *in vitro*, we performed EMSA using recombinant HbMADS4 protein (**Figure 6A**) and 1050-bp *HbSRPP* promoter nucleotide probes. As shown in **Figure 6B,C**, HbMADS4 was able to bind with *HbSRPP* promoter and cause mobility shifts. Moreover the quantity of shifted protein-DNA complexes increased gradually, and the quantity of free DNA levels decreased accordingly (**Figure 6B**, lanes 2–6). The results showed that *in vitro* HbMADS4 was able to recognize and interact with the *HbSRPP* promoter.

### Suppression of the *HbSRPP* Promoter by HbMADS4

**FIGURE 7:**
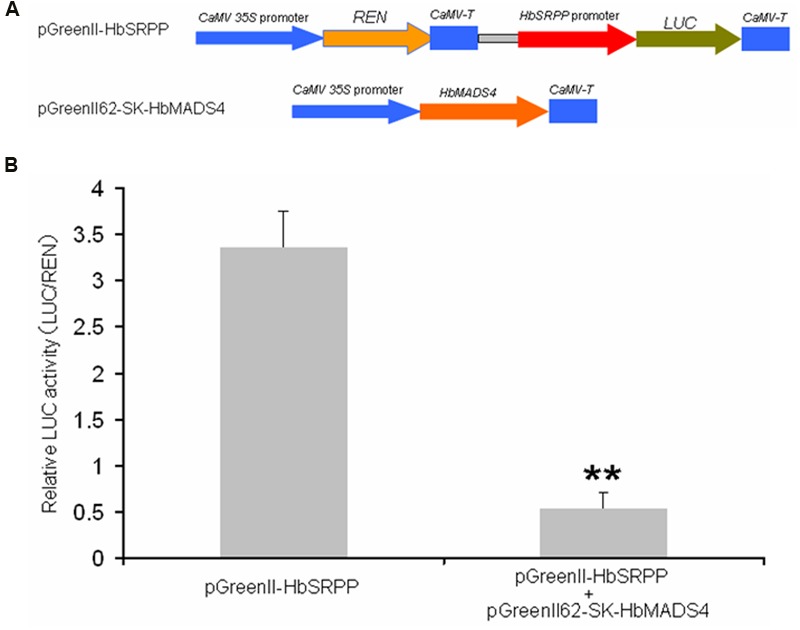
**Repression of *HbSRPP* promoter in transient expression system by HbMADS4. (A)** Schematic diagrams of the transient expression vectors used in the transient expression analysis. **(B)** Relative LUC activity from transient expression analysis of *HbSRPP* promoter co-infiltrated with a plasmid containing gene for HbMADS4 fused to the 35S promoter.

Given the fact that *in vitro* HbMADS4 was able to interact with the *HbSRPP* promoter, we asked if HbMADS4 can regulate the *HbSRPP* promoter in plant. A dual-luciferase assay system was employed for this purpose (**Figure 7A**). The level of the luciferase activity controlled by HbMADS4 and *HbSRPP* promoters was elevated (**Figure 7B**), the expression of *HbMADS4* resulted more than 6 folds decrease of the luciferase activity. The result shows that transient over-expression of *HbMADS4* suppressed the *HbSRPP* promoter.

**FIGURE 8:**
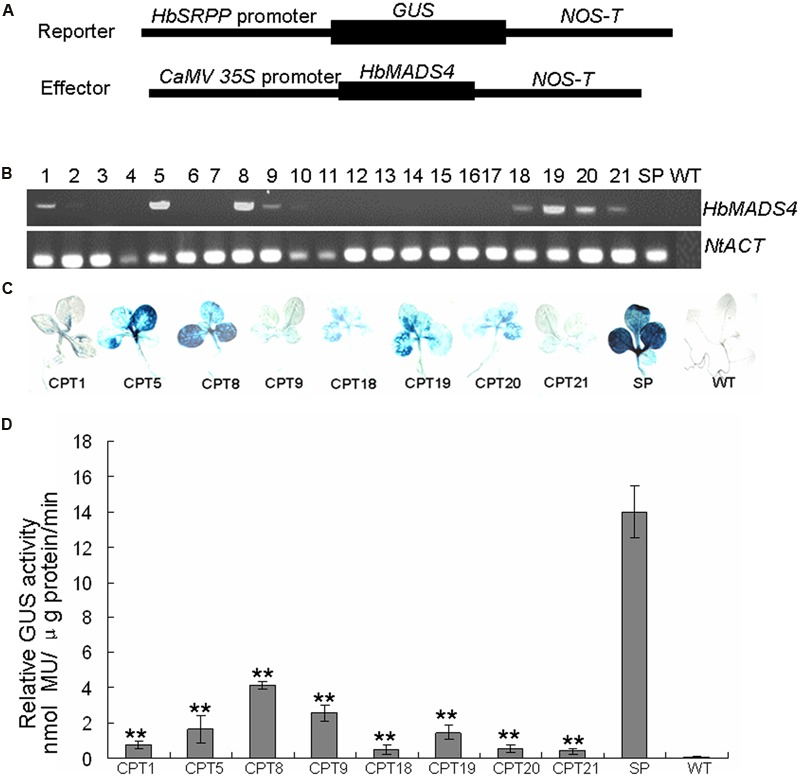
**Repression of *HbSRPP* promoter in transgenic plants by HbMADS4. (A)** Schematic diagrams of the reporter and effector constructs used in the experiment. **(B)** The co-transformed plants (CTPs) were examined by RT-PCR. **(C)** Histochemical staining analysis of the different CTPs (i.e., CPT1, 5, 8, 9, 18, 19, 20, and 24), wild-type (WT) and plant transformed with p*HbSRPP*::*GUS* (PH) were used as controls. **(D)** Quantitative determination of the GUS activities in the different cCTPs (i.e., CPT1, 5, 8, 9, 18, 19, 20, and 24), WT and PH were used as controls. Three replicates were included for each sample. Data are presented as mean ± SE (*n* = 3), and the significant difference was assessed by ANOVA (two asterisks corresponding to *P* < 0.01).

It is very difficult to obtain transgenics in rubber tree. To further test whether HbMADS4 can regulate the *HbSRPP* promoter, tobacco plants were co-transformed with the vector carrying the *HbMADS4* cDNA fused to the CaM 35S promoter, and a PH construct (**Figure 8A**). Plants transformed with PH and CTPs with PH and *CaMV35S::HbMADS4* were selected by hygromycin and kanamycin resistance, CTPs were further detected by RT-PCR (**Figure 8B**) and GUS histochemical staining (**Figure 8C**). Over-expression of *HbMADS4* suppressed the *HbSRPP* promoter in the CTPs compared with in the PH. As shown in **Figure 8D**, in CTPs (i.e., CTP1, 5, 8, 9, 18, 19, 20, and 21), expression of *HbMADS4* resulted in a more than 4 folds decrease of *HbSRPP* promoter activity. The results show that the over-expression of HbMADS4 strongly suppressed the *HbSRPP* promoter.

## Discussion

MADS-box genes play important roles during plant growth and development, especially floral organ and fruit development. To date, many plant MADS-box genes have been identified in controlling reproductive development processes (Smaczniak et al., 2012a,b). Three MADS-box genes had been isolated from *H. brasiliensis*. The three genes were differentially expressed during somatic embryogenesis of rubber tree (Li et al., 2011). In this study, *HbMADS4* was isolated from *H. brasiliensis* by the yeast one-hybrid experiment to screen the latex cDNA library using the *HbSRPP* promoteras bait. *HbMADS4* was preferentially expressed in latex, but little in leaves, roots and flower. Laticifers are of importance in NR production since within them NR is formed and stored. Genes highly expressed in the laticifers maybe involved in rubber synthesis (Oh et al., 1999; Han et al., 2000). *HbMADS4* was preferentially expressed in latex implies that active expression of *HbMADS4* might play important role in rubber synthesis.

HbSRPP is a major rubber particles protein of 22.4 kD in the latex (Yeang et al., 1996; Oh et al., 1999). HbSRPP has been suggested to be tightly associated with small rubber particles and also involved in rubber biosynthesis (Berthelot et al., 2012). HbSRPP was susceptible to play a role in latex coagulation (Wititsuwannakul et al., 2008). *HbSRPP* is highly expressed in the laticifers (Han et al., 2000; Ko et al., 2003) and is regulated by MeJA, ABA, GA, cold, heat, and wounding (Guo et al., 2014). HbWRKY1, a WRKY TF, was found to negatively regulate *HbSRPP* expression (Wang et al., 2013). WRKY TFs specifically bind to the W-box (T)TGAC(C/T), which contains the invariant TGAC core (Ciolkowski et al., 2008). There are three W-boxes at position +60, +84, and +487 in the *HbSRPP* promoter region. The MADS TFs binds to the CArG-box (West et al., 1998). There are two CArG-boxes at position +502 and +702 in the *HbSRPP* promoter region. HbMADS4 interacts with the *HbSRPP* promoter *in vitro* and suppress the activity of the *HbSRPP* promoter in transgenic tobacco. The results strongly indicate that HbMADS4 is a transcriptional suppressor of *HbSRPP*. It will be of great interest to elucidate *HbSRPP* is regulated by MADS-box TFs in rubber tree.

## Conclusion

A novel MADS-box TF gene, designated as *HbMADS4*, was isolated from *H. brasiliensis HbMADS4* was preferentially expressed in the latex, but little expression was detected in the leaves, flowers, and roots. Its expression was inducible by MeJA and ethylene. HbMADS4 bound to the *HbSRPP* promoter. Over-expression of HbMADS4 in transgenic tobacco plants significantly suppressed the activity of the *HbSRP* promoter. Altogether, it is proposed that HbMADS4 is a negative regulator of *HbSRPP* which participating in the biosynthesis of NR.

## Author Contributions

H-LL and S-QP designed the research, L-RW, H-LL, DG, YW, J-HZ, and X-TC performed the research, and HL-L and S-QP wrote the paper. All authors read and approved the final manuscript.

## Conflict of Interest Statement

The authors declare that the research was conducted in the absence of any commercial or financial relationships that could be construed as a potential conflict of interest.
